# Supplementary material for: Two-dimensional antimonene single crystals grown by van der Waals epitaxy
Source: Nat Commun. 2016 Nov 15;7:13352. doi: 10.1038/ncomms13352 (PMC5116078; doi:10.1038/ncomms13352)
Supplement: Supplementary Information — Supplementary Figures 1-11. [file ncomms13352-s1.pdf]

Supplementary Figures

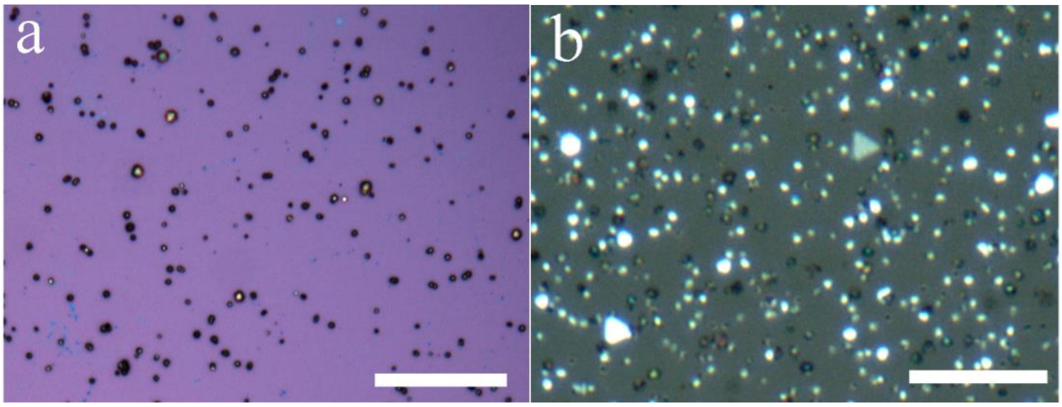

Supplementary Figure 1. Optical microscope images of antimonene polygons grown on (a) silicon substrate and (b) sapphire substrate. The scale bars are 5 μm.

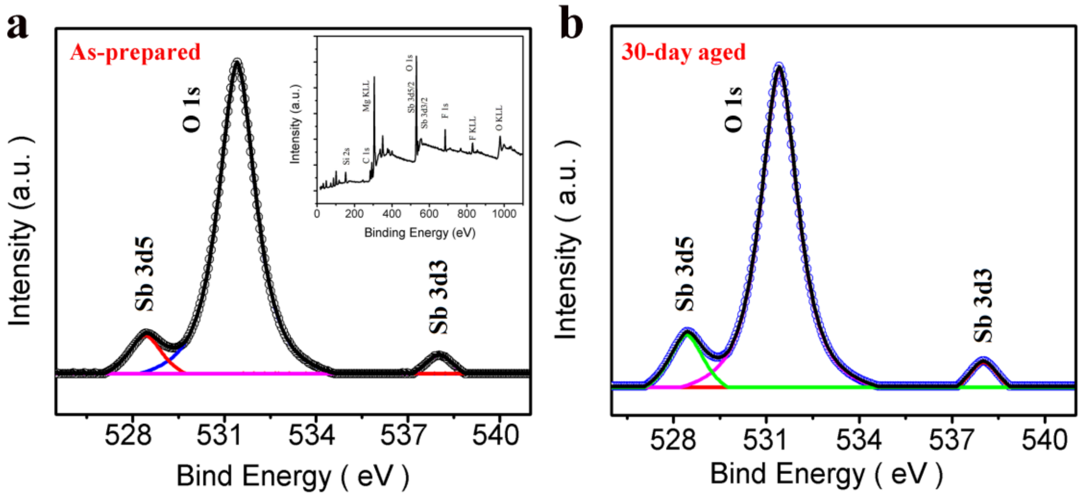

Supplementary Figure 2. XPS survey spectra of Sb/mica sample. (a) Sb 3d region spectrum of one freshlt-made sample. In Sb 3d region, 3d3 and 3d5 peaks at 528.4 eV and x538 eV, respectively. The O 1s peak is attributed to mica. (b) Sb 3d region spectrum of one sample stored for 30 days. No obvious peak shift is observed. The different intensity ratio of Sb 3d and O1s in Figure a and b is attributed to different coverage in the test zone randomly selected.

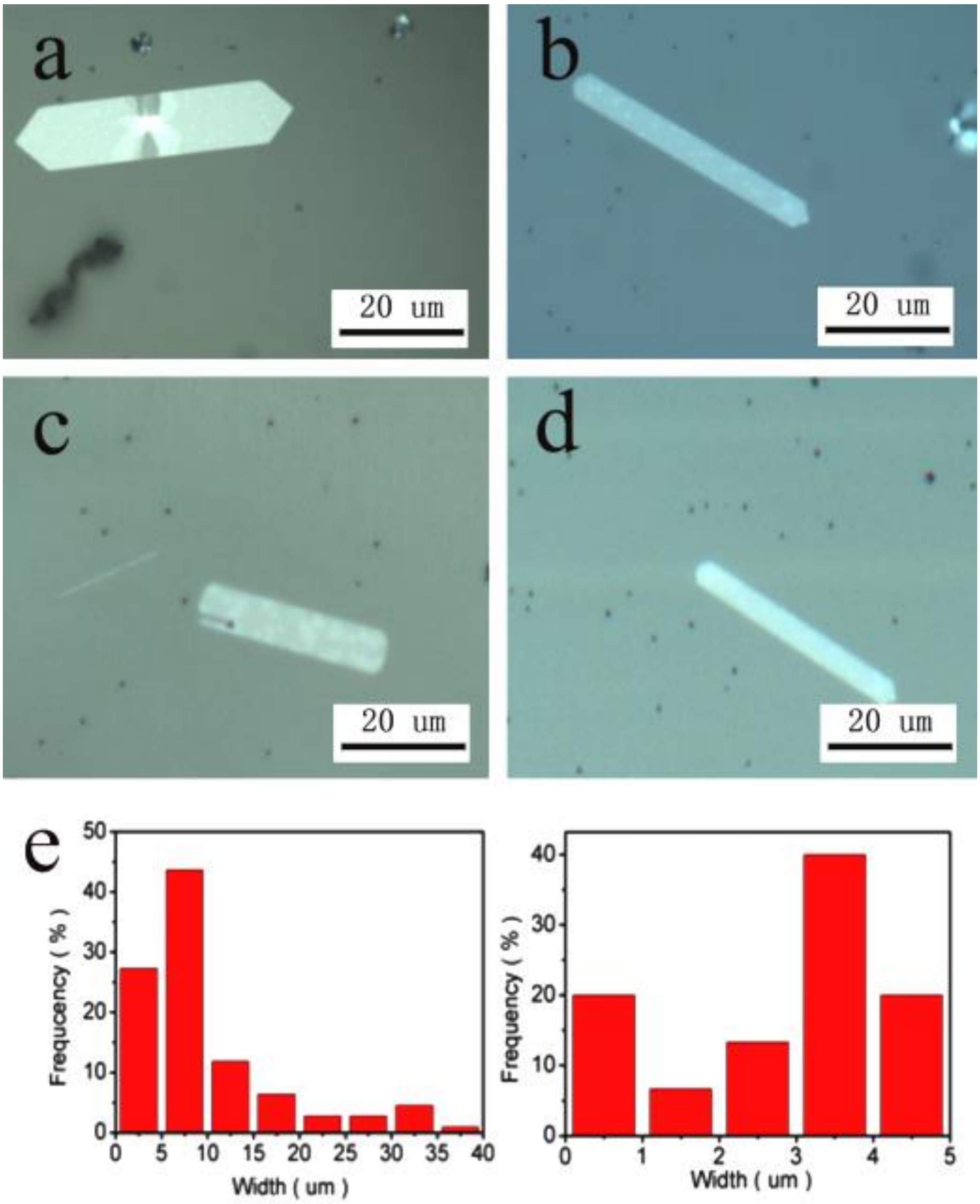

Supplementary Figure 3. Optical microscope image of several typical antimonene sheets with stretched hexagonal shape. (a-d) Similar stretched hexagonal polygons could be mistaken as ribbons when the lateral sizes are small or when the image magnification is low. (e) Despersion of ribbons width ranging from 0 to 40 um(left) and 5um (right).

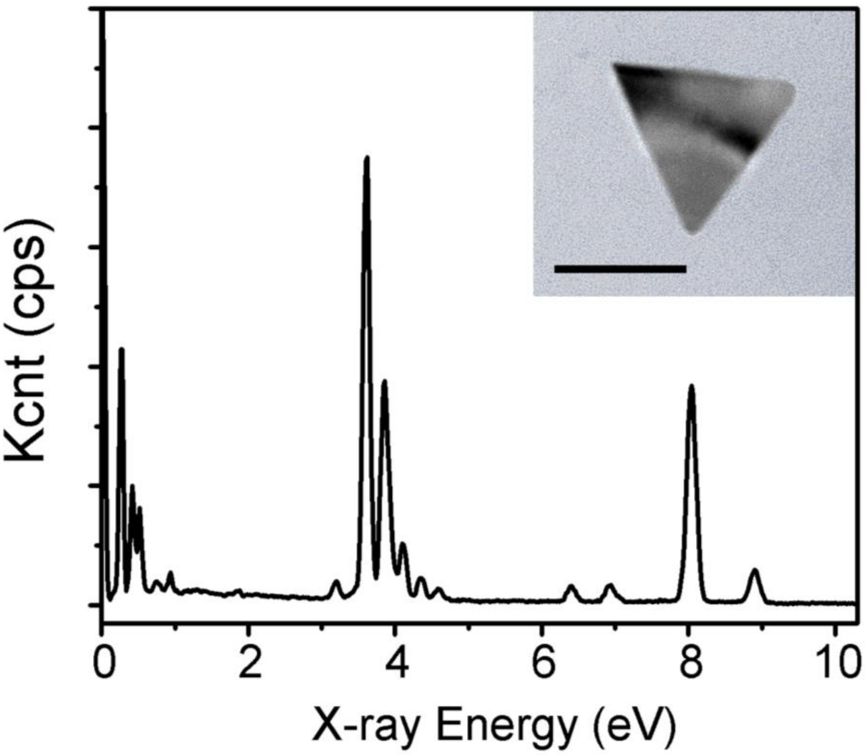

Supplementary Figure 4. EDS spectrum of one tiny nanosheet. The EDS spereum indicates the tiny sample composed of antimony. The scale bar(inset) is 100 nm.

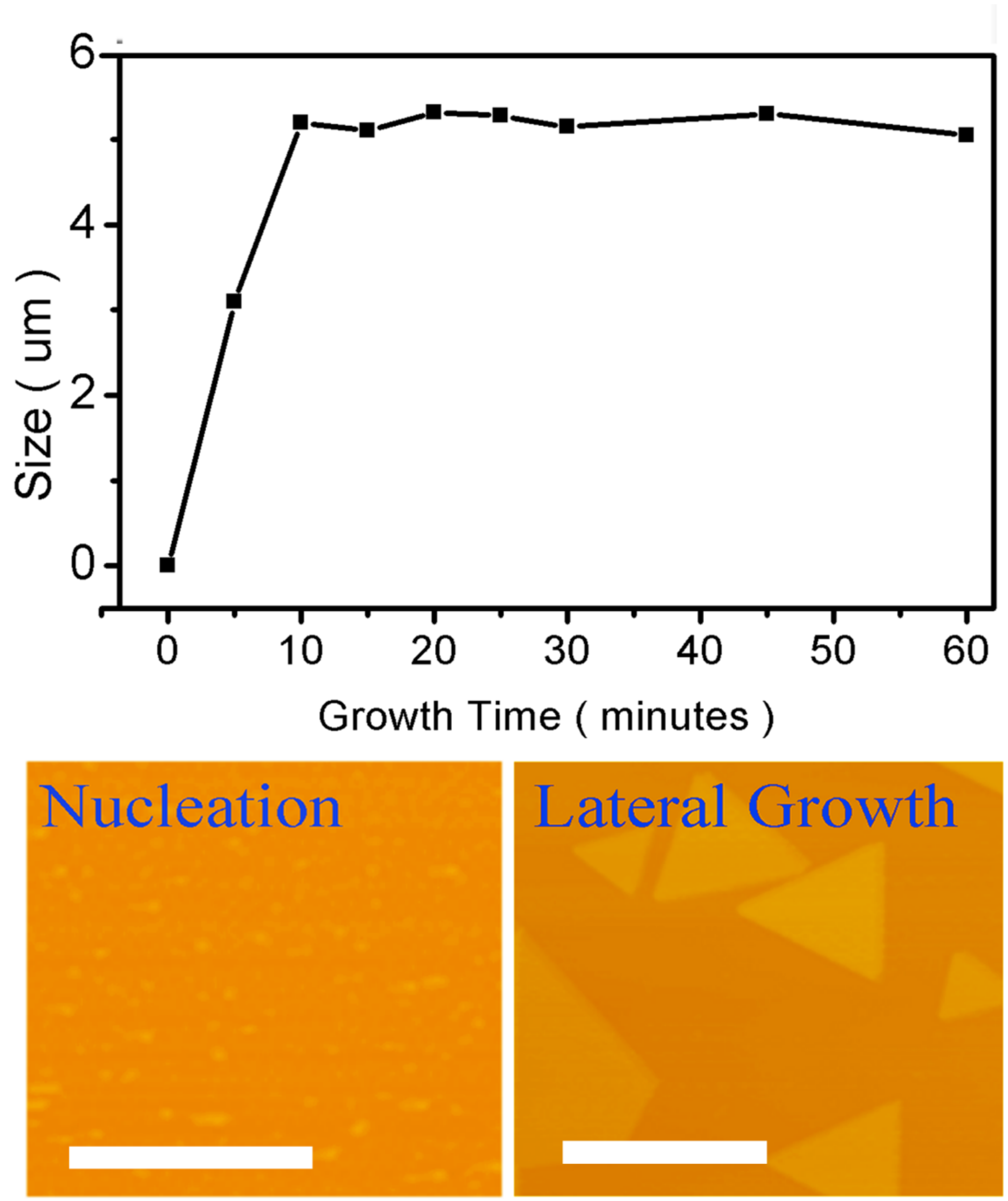

Supplementary Figure 5. Lateral size as a function of growth time and AFM images of nucleation and lateral growth. Antimonene layers finished growth in the front 10 minutes, even for longer dutations. The scale bars of the nucleation and growth are 500 nm and 5 μm, respectively.

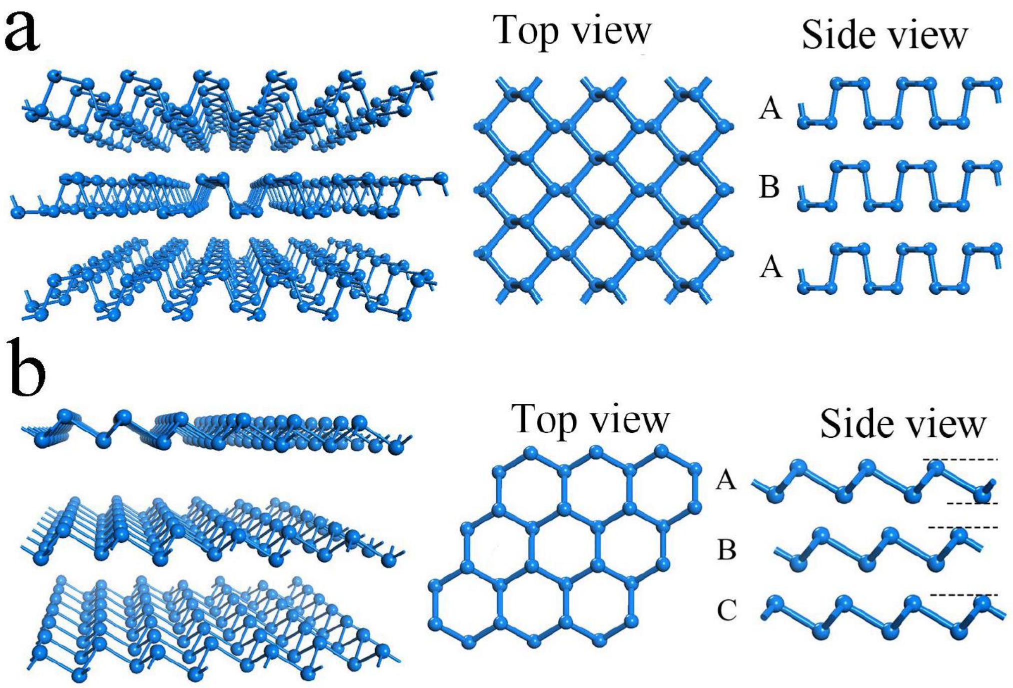

Supplementary Figure 6. The comparison of atomic structures of α-phase (a) and β-phase (b) antimony. α-phase antimony has orthorhombic structure and β-phase antimony has rhombohedral structure.

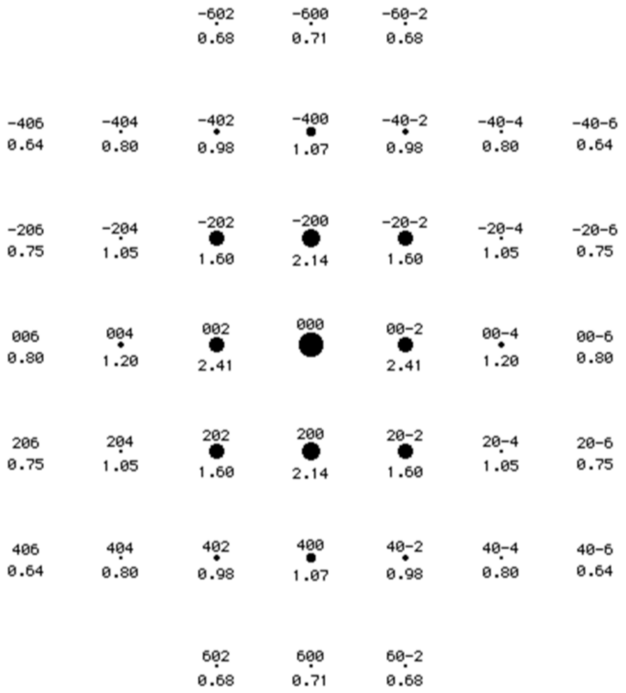

α-phase, along [001] zone axis

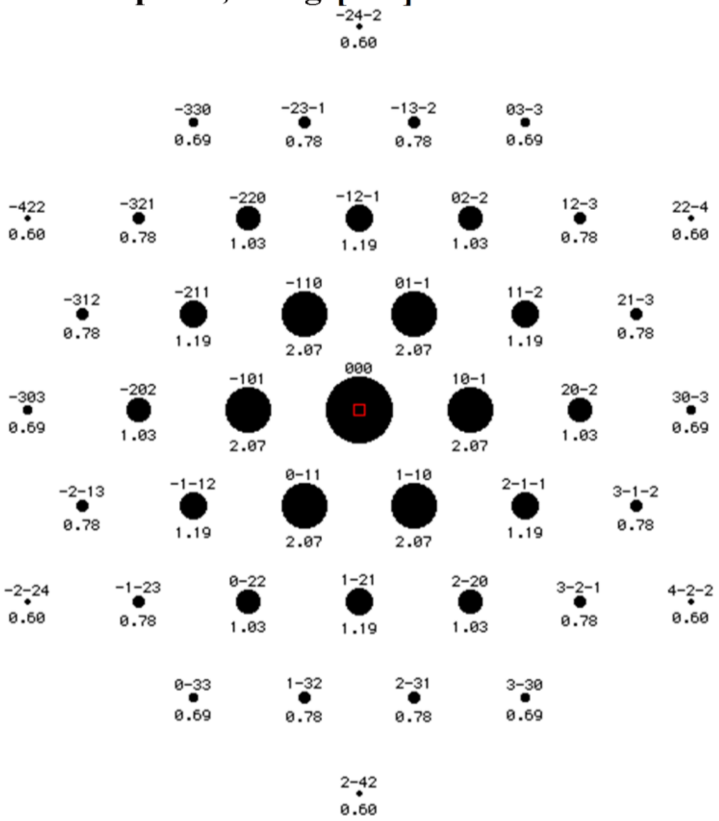

β-phase, along [001] zone axis

Supplementary Figure 7. The simulated kinematical SAED pattern of α-phase and β-phase .

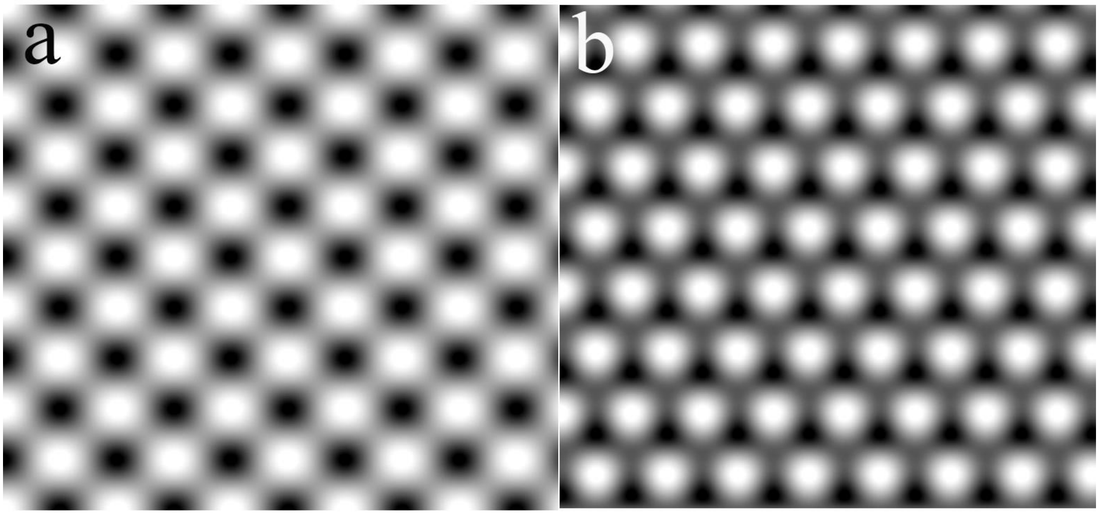

Supplementary Figure 8. Simulated HRTEM images of α-phase (along [001] zone axis) and β-phase (along [001] zone axis) few-layer antimonenes using the same microscopy parameters.

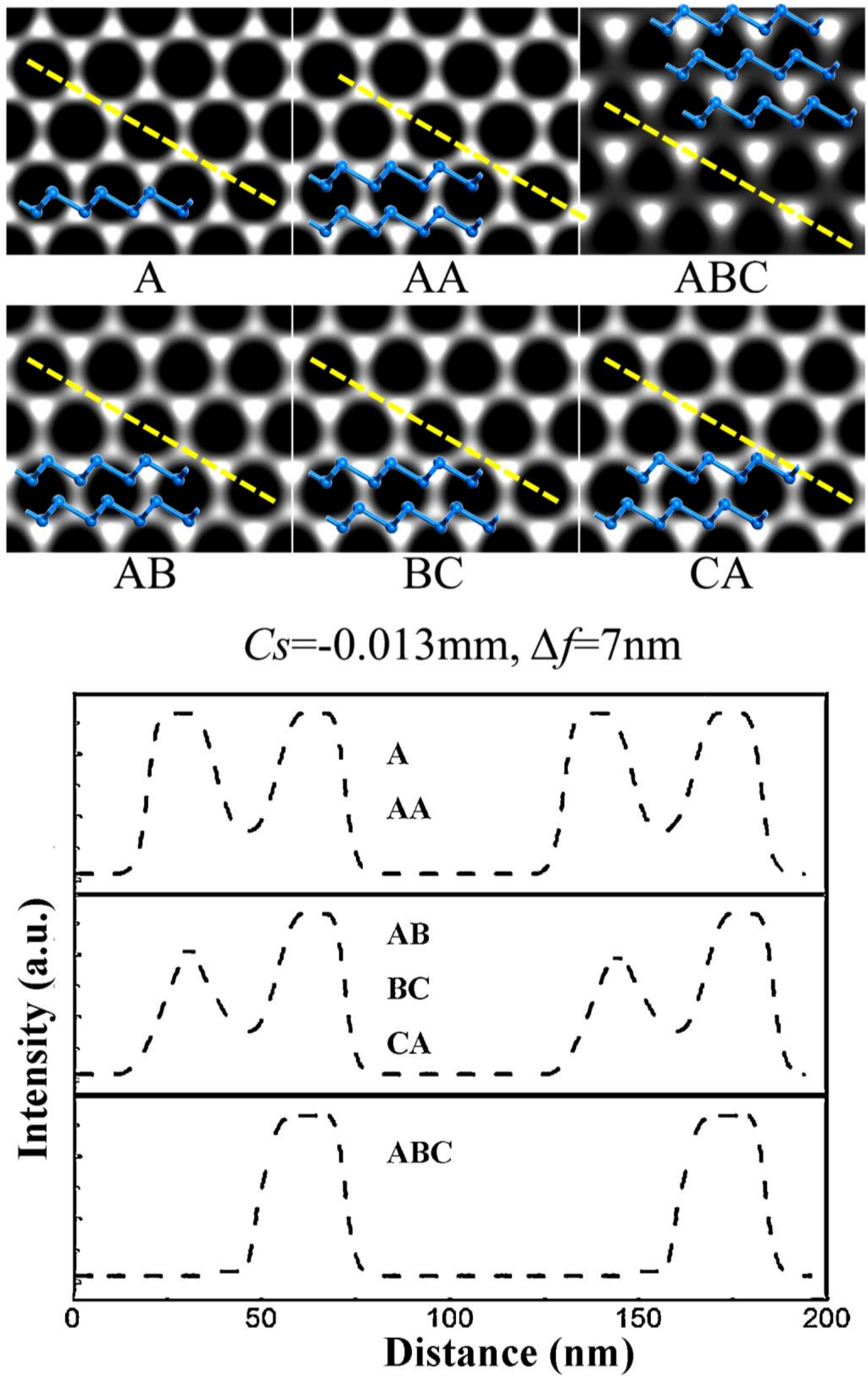

Supplementary Figure 9. Simulated atomic images of antimonene with different stacking under the the condition of Cs = -13 µm and ΔZ = +7 nm and dash scanning files of different stacking order.

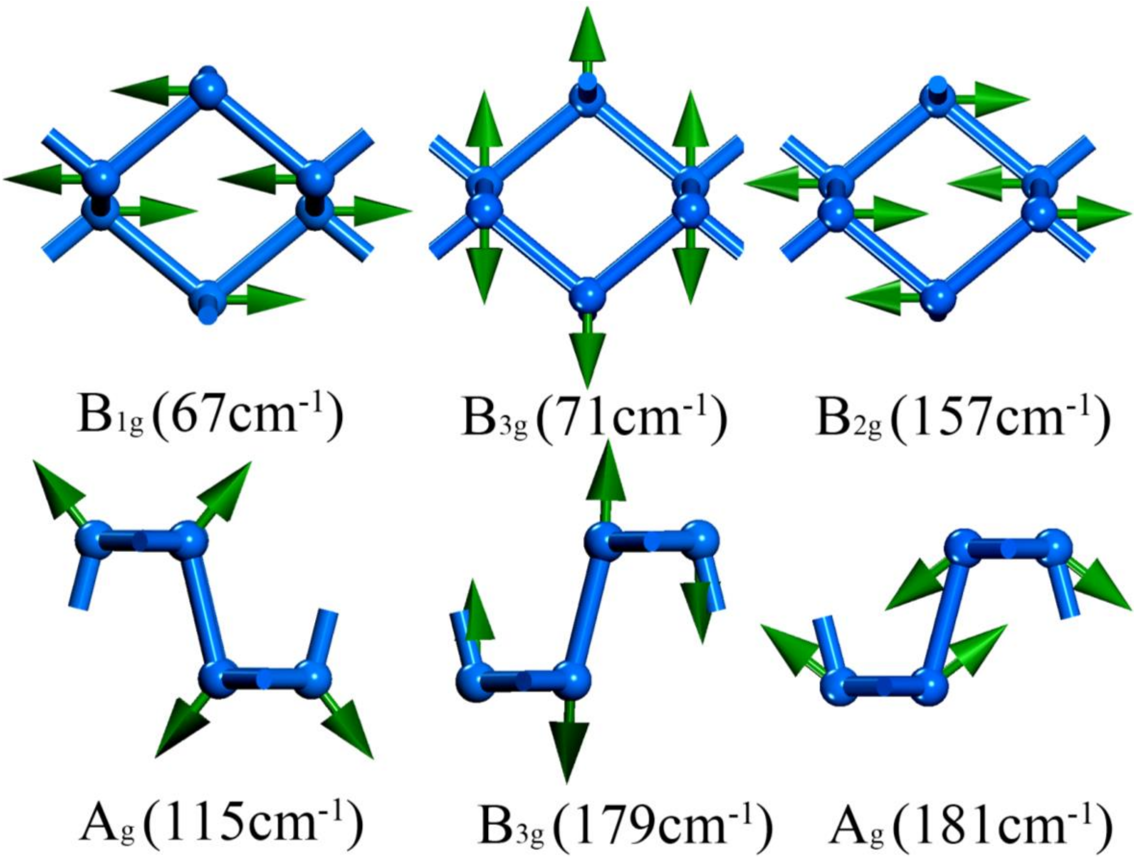

Supplementary Figure 10. Raman active modes of α-phase monolayer antimonene. The frequencies are obtained from the first-principle calculations with the LDA method.

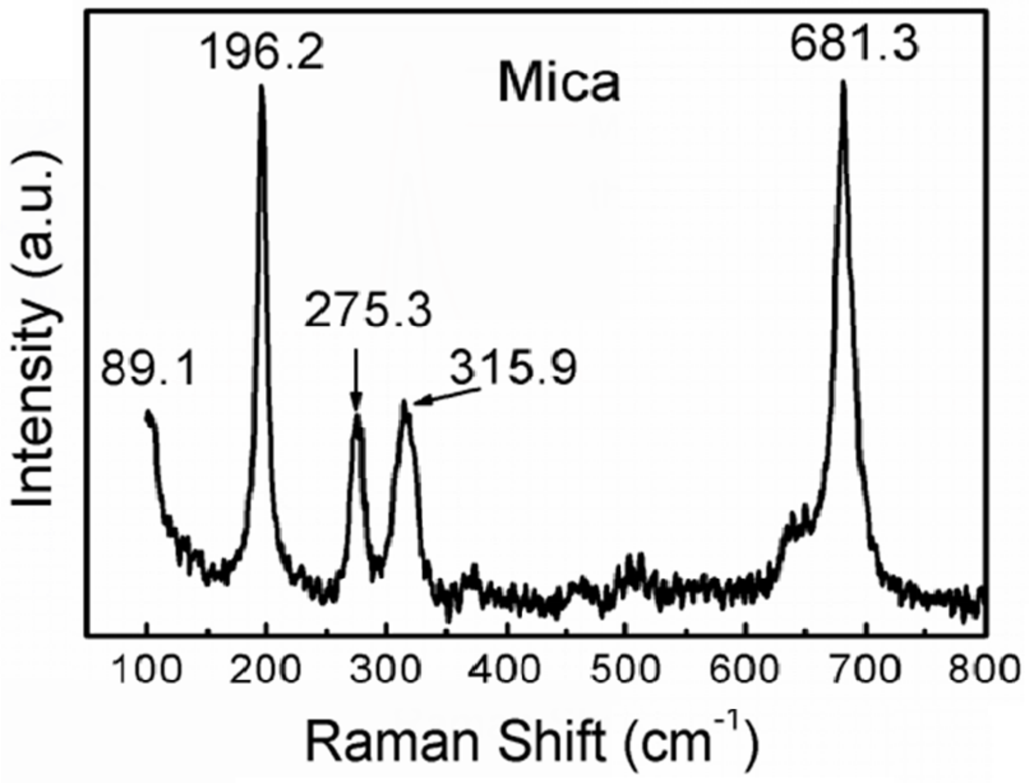

Supplementary Figure 11. Raman spectrum of mica substrate measured under the same conditions as the Raman measurement of antimonene polygons.
